# Pre-Pregnancy Weight Status Is Associated with Diet Quality and Nutritional Biomarkers during Pregnancy

**DOI:** 10.3390/nu8030162

**Published:** 2016-03-11

**Authors:** Dayeon Shin, Kyung Won Lee, Won O. Song

**Affiliations:** Department of Food Science and Human Nutrition, Michigan State University, 469 Wilson Road, G. Malcolm Trout FSHN Building, East Lansing, MI 48824, USA; shinda@msu.edu (D.S.); kyungwon@msu.edu (K.W.L.)

**Keywords:** diet quality, Healthy Eating Index (HEI)-2010, pre-pregnancy body mass index (BMI), nutritional biomarkers, National Health and Nutrition Examination Survey

## Abstract

Although the positive association between pre-pregnancy overweight and obesity with excessive gestational weight gain is well known, it is not clear how pre-pregnancy weight status is associated with gestational weight gain through maternal diet during pregnancy. This study aimed to examine the relationship between pre-pregnancy weight status and diet quality and maternal nutritional biomarkers during pregnancy. Our study included 795 U.S. pregnant women from the National Health and Nutrition Examination Survey, 2003–2012. Pre-pregnancy body mass index (BMI) was calculated based on self-reported pre-pregnancy weight and height. The cutoff points of <18.5 (underweight), 18.5–24.9 (normal), 25.0–29.9 (overweight), and 30 kg/m^2^ (obese) were used to categorize pregnant women’s weight status. Diet quality during pregnancy was assessed by the Healthy Eating Index (HEI)-2010 based on a 24-h recall. Multivariable logistic regressions were used to estimate the odds ratios (OR) and 95% confidence intervals (CI). For all pregnant women included in this study, the mean HEI-2010 (±standard error of the mean (SEM)) was 50.7 (±0.9). Women with obese pre-pregnancy BMI demonstrated significantly lower HEI-2010 compared to those with underweight and normal pre-pregnancy BMI, respectively. In an unadjusted model, women with pre-pregnancy obesity BMI had increased odds for being in the lowest tertile of HEI-2010 (33.4 ± 0.5) compared to those with underweight pre-pregnancy BMI (OR 5.0; 95% CI 2.2–11.4). The inverse association between pre-pregnancy overweight and obesity status and diet quality during pregnancy persisted even after we controlled for physical activity levels (adjusted OR (AOR) 3.8; 95% CI 1.2–11.7, AOR 5.4; 95% CI 2.0–14.5, respectively). Serum folate concentration (ng/mL) was significantly higher in underweight women compared to overweight women (23.4 ± 1.7 *vs.* 17.0 ± 0.8, *p* < 0.05). Serum iron concentration (ng/dL) was significantly higher in normal weight women compared to overweight women (86.2 ± 5.0 *vs.* 68.9 ± 3.0, *p* < 0.05). An inverse association was found between pre-pregnancy weight status and diet quality and maternal nutritional biomarkers during pregnancy. Poor diet quality as measured by HEI-2010 was shown among overweight and obese women. Nutrition education and interventions need to be targeted to those women entering pregnancy as overweight and obese.

## 1. Introduction

Maternal diet before and during pregnancy may play an important role in maternal, neonatal, and child health outcomes [1,2]. Specifically, pregnant women require additional folate and iron before and during pregnancy to meet their own needs and optimize birth outcomes [3]. Preterm birth and low birth weight have been associated with inadequate folate intake [4] and iron deficiency during pregnancy [5]. In the United States, women of childbearing age who are overweight or obese had lower serum concentrations of folate compared to those women with underweight and normal weight [6]. Adverse birth outcomes have been associated with pre-pregnancy overweight and obesity and inadequate maternal nutrition during pregnancy.

Overweight and obesity status before pregnancy has been found to be associated with excessive gestational weight gain [7], which in turn is associated with postpartum weight retention [8]. Pre-pregnancy weight status has also been reported as an independent determinant for gestational diabetes mellitus, gestational hypertension, preterm birth, and small and large for gestational age births in U.S. pregnant women [9]. Diet during pregnancy may partially mediate the relationship between pre-pregnancy overweight and obesity and pregnancy complications and birth outcomes [10]. Laraia *et al.* [11] first demonstrated that pre-pregnancy BMI was inversely associated with diet quality as measured by the Diet Quality Index for Pregnancy (DQI-P) in pregnant women in North Carolina. In a cross-sectional study of Greek women, Tsigga *et al.* [12] also reported that pregnant women who were underweight or normal weight before pregnancy demonstrated a better diet quality as assessed by the Healthy Eating Index (HEI)-2005 compared to women with obese pre-pregnancy BMI. However, the majority of the study population in these studies [11,12] were low- to middle-income non-Hispanic white women. Thus, this may not be representative of the entire population of U.S. pregnant women.

Gestational weight gain guidelines were established by the Institute of Medicine for the optimal health outcomes for the mother and the offspring [13]. The guidelines are based on pre-pregnancy weight status as the obesity epidemic has been increasing among reproductive aged women in the United States [13]. It is unclear how pre-pregnancy weight status is associated with gestational weight gain through maternal diet during pregnancy. Diet during pregnancy may play a significant role linking the association between pre-pregnancy weight status and gestational weight gain. Recently, Graziano *et al.* [14] found that HEI-2010 may provide useful information assessing overall diet quality during pregnancy. It is important to examine the relationship between pre-pregnancy weight status and diet quality assessed by HEI-2010 and nutritional biomarkers including serum folate, iron, and ferritin concentrations during pregnancy in U.S. representative pregnant women.

## 2. Material and Methods

### 2.1. Study Population

We used public domain data from the continuous National Health and Nutrition Examination Survey (NHANES) 2003–2004, 2005–2006, 2007–2008, 2009–2010, and 2011–2012 for this study. NHANES is a program of studies cross-sectionally designed to assess the health and nutritional status of civilian, non-institutionalized population in the United States conducted by the National Center for Health Statistics (NCHS), Centers for Disease Control and Prevention (CDC). The NHANES used a stratified multistage probability sample that was based on the selection of counties, blocks, households, and finally persons within households. The NHANES survey is unique in that it combines interviews and physical examinations. The participants were interviewed for the information of age, race/ethnicity, education level, marital status, family poverty income ratio, and physical activity. Reproductive health interviews obtained information on month of gestation at the time of the survey. Pregnancy status was based on a positive urine pregnancy test or self-reported pregnancy. A complete description of data-collection procedures and analytic guidelines has been provided elsewhere [15,16].

The 2003–2012 NHANES dataset included 856 pregnant women. Subjects were excluded if they reported unreliable dietary data, as defined by the NCHS [17]. Included in the present study were participants with complete data for all variables of interest: pregnancy urine test, age, race/ethnicity, family poverty income ratio, education, marital status, trimester of pregnancy, self-reported pre-pregnancy weight, and measured height and weight. The final analytic sample size was 795 pregnant women.

### 2.2. Exposure Variable

Self-reported pre-pregnancy weight and measured height were used to calculate pre-pregnancy BMI. We have previously demonstrated that pre-pregnancy weight status classified based on self-reported pre-pregnancy height and weight was valid [18]. We found that mean (SEM) differences between self-reported pre-pregnancy weight *versus* measured weight in the first trimester was –2.3 (0.7) kg with *r* = 0.98 (*p* < 0.001) and κ = 0.76, which also showed substantial agreement in 95 pregnant women. Self-reported pre-pregnancy weight status was stratified into four categories based on the WHO criteria [19]: <18.5 kg/m^2^ (underweight), 18.5–24.9 kg/m^2^ (normal), 25.0–29.9 kg/m^2^ (overweight), and ≥30 kg/m^2^ (obese).

### 2.3. Outcome Variables

Dietary intake was measured via an in-person 24-h recall collected by trained personnel of National Center for Health Statistics (NCHS) using the USDA’s Automated Multiple-Pass Method [20]. The HEI is a measure of diet quality in conformance to the Dietary Guidelines for Americans, which are the basis of nutrition policy for the U.S. government and the foundation of all federal nutrition guidance [21]. The HEI-2010 is made up of 12 components: nine adequacy components (total fruit; whole fruit; total vegetables; greens and beans; whole grains; dairy; total protein foods; seafood and plant protein; and fatty acids) and three moderation components (refined grains; sodium; and empty calories) (Table 1) [21]. For the adequacy component, a higher score corresponds to a higher intake. For the moderation component, a higher score corresponds to lower intake. The total HEI-2010 scores range from 0 (non-adherence) to 100 (perfect adherence). The MyPyramid Equivalent Database (MPED) 2.0, Food Patterns Equivalents Database (FPED) 2005–2006, FPED 2007–2008, FPED 2009–2010, and FPED 2011–2012 with the addendum from the Center for Nutrition Policy and Promotion was used for food grouping [22]. The scoring method of the HEI-2010 is described elsewhere [21] and summarized in Table 1. In our study, a categorical variable was created using the HEI-2010 tertiles as cut-off points to compare the lowest with the highest tertile as a reference.

Daily energy intake, percent energy from carbohydrates, protein and intakes of fat, folate, iron, and calcium from one-day 24-h recall were calculated. Two 24-h dietary recalls were collected by two different dietary collection methods: day 1 by interview by trained dietary interviewers in the Mobile Examination Center *vs.* day 2 by telephone interview. To avoid the bias resulting from data collection methods, we used one day’s dietary intake rather than an average of two days. Nutritional biomarkers including total iron concentrations were obtained from the NHANES standard biochemistry profile dataset. Detailed descriptions and instructions can be found in the NHANES Laboratory/Medical Technologists Procedures Manual [23]. Briefly, serum concentrations of total iron were measured by the DxC800 System [24]. Serum folate and ferritin concentrations were also assessed in relation to pre-pregnancy weight status. Serum folate concentration was measured by using the Quantaphase II (Bio-Rad Laboratories, Hercules, CA, USA) during NHANES 2003–2006 [25] and NHANES 2007–2010 [26], and by the isotope-dilution high performance liquid chromatography coupled to tandem mass spectrometry during NHANES 2011–2012 [27]. Ferritin was measured by immune turbidimetry using a Roche/Hitachi 912 clinical analyzer [28].

### 2.4. Covariates

Analyses were adjusted for maternal age, race/ethnicity, family poverty income ratio, education, marital status, smoking status, and physical activity level. Maternal age was divided into three groups: ≤24, 25–34, and ≥35 years. The study group consisted of Mexican-American or other Hispanic, non-Hispanic white, non-Hispanic black, or other races. Family poverty income ratio, defined as the ratio of family income in relation to the 100% poverty guidelines for various family sizes as determined by the Department of Health and Human Services [29], was divided into three categories: ≤1.85, 1.85–4, and >4. Maternal education was grouped by the number of completed years of school: less than high school, high school diploma, and more than high school. Marital status was divided into three groups: married, widowed/divorced/separated/living with a partner, and single. Smoking status was defined by serum cotinine concentrations (non-smoker: ≤10 mg/L; smoker >10 mg/L). Physical activity level was divided into four groups: no activity, 0–500 MET-min/week, 500–1000 MET-min/week, and ≥1000 MET-min/week.

### 2.5. Statistical Analyses

Descriptive statistics for the main variables of interest were generated. Analysis of variance with Bonferroni correction was conducted for each of the 12 HEI-2010 components and overall HEI-2010 scores across the categories of pre-pregnancy weight status.

A multivariable linear regression model was used to examine the association of maternal sociodemographic factors and physical activity levels with HEI-2010 as a continuous variable. The overall HEI-2010 scores were categorized into tertiles using the highest tertile as a reference group. A multivariable logistic regression model was used to estimate the association of pre-pregnancy weight status with the lowest tertile of the HEI-2010. We ran models in three ways: (1) crude; (2) model 1: adjusted for age, race/ethnicity, family poverty income ratio, education, marital status, and smoking status; and (3) adjusted for model 1 + physical activity.

To analyze the magnitude of collinearity among covariates, the variance inflation factor (VIF) was used to test with VIF <5 set as the acceptable level [30]. We accounted for the stratified, multi-stage probability design used in NHANES 2003–2012. Appropriate sample weights were applied in all statistical analyses to produce estimates of means and percentiles that can be generalized to the healthy U.S. adult population. All analyses were carried out using SAS software (version 9.3; SAS Institute, Cary, NC, USA). A *p*-value < 0.05 was declared as statistically significant.

## 3. Results

Pregnant women included in this study were 52% non-Hispanic white, 23% Mexican American or other Hispanic, 18% non-Hispanic black, and 8% other race; 64% were married; and 91% had between 1 and 5 previous live births. Forty-four percent had an income of <185% of the poverty level (the income eligibility criterion for the Special Supplemental Nutrition Program for Women, Infants, and Children (WIC)). Fifty-nine percent had more than a college level education, 39% were in their third trimester of pregnancy, 9% were smokers during pregnancy, and 35% engaged in light leisure-time physical activities during pregnancy (Table 2).

For all pregnant women included in this study, the mean HEI-2010 (±standard error of the mean (SEM)) was 50.7 (±0.9). The mean HEI-2010 score varied significantly by maternal sociodemographic characteristics (Table 2). Significantly higher mean HEI-2010 scores were found for pregnant women who were older than 35, other race including multi-racial groups, family poverty income ratio above 4, married, and non-smokers. Multi-collinearity between age, race/ethnicity, family poverty income ratio, education, marital status, parity number, trimester of pregnancy, smoking status, and physical activity were assessed. The VIF for all the confounding variables ranged from 1.04 to 1.59. These findings suggest that collinearity between these confounding variables was not significant.

Table 3 shows covariate-adjusted mean HEI across all the pre-pregnancy weight status groups. After adjusting for maternal age, race/ethnicity, family poverty income ratio, education, marital status, smoking status and physical activity, women with obese pre-pregnancy BMI had significantly lower overall HEI-2010 compared to those with normal pre-pregnancy BMI (48.8 ± 2.0 *vs.* 55.2 ± 1.6). Women with obese pre-pregnancy BMI had significantly lower scores for the sodium component compared to normal weight women (3.7 ± 0.6 *vs.* 5.4 ± 0.4) (Table 3).

Table 4 represents mean values for dietary intake and diet-related biomarkers across pre-pregnancy weight status groups. Intakes of folate (mcg)/1000 kcal and iron (mg)/1000 kcal significantly differed by pre-pregnancy weight status. Women of obese pre-pregnancy BMI had significantly lower intake of both folate and iron per 1000 kcal compared to women of underweight pre-pregnancy BMI. Serum folate (ng/mL) and iron (ug/dL) concentrations were significantly differed by pre-pregnancy weight status groups. Serum folate concentration was significantly higher in underweight women compared to overweight women (23.4 ± 1.7 *vs.* 17.0 ± 1.8 ng/mL). Serum iron concentration was significantly higher in normal weight women compared to overweight women (86.2 ± 5.0 *vs.* 68.9 ± 3.0 ug/dL). Folic acid, iron, zinc, and calcium intakes from dietary supplements did not differ by pre-pregnancy weight status (Table 4).

In the unadjusted logistic regression analysis, results show that women with pre-pregnancy overweight and obese BMI had increased odds of falling into the lowest (mean 33.4 ± SEM 0.5) *vs.* the highest (mean 66.5 ± SEM 0.9) HEI-2010 tertile compared with underweight BMI (OR 2.6; 95% CI 1.1–6.4, OR 5.0; 95% CI 2.2–11.4, respectively) (Table 5). We then compared two models controlling first for maternal age, race/ethnicity, family poverty income ratio, education level, marital status, and smoking status. In the second model, we controlled for the covariates controlled in the first model as well as leisure-time physical activity level during pregnancy. The inverse association between pre-pregnancy overweight and obesity and diet quality during pregnancy remained significant after we adjusted for maternal characteristics (adjusted OR (AOR) 2.8; 95% CI 1.2–6.6, AOR 3.7; 95% CI 1.7–8.2). The inverse association between pre-pregnancy overweight and obesity and diet quality persisted even after we controlled for physical activity levels (AOR 3.8; 95% CI 1.2–11.7, AOR 5.4; 95% CI 2.0–14.5) (Table 5).

## 4. Discussion

Recently, HEI-2010 has been reported to be valid and reliable in assessing the overall diet quality of an individual in terms of conforming to federal dietary guidance [31]. However, Pick *et al.* [32] reported that the HEI was useful in providing a composite measure of dietary intake, but did not discern the need for vitamin and mineral supplements during pregnancy. To overcome this issue, we examined folate, iron, and calcium from both a dietary recall and their biomarker values across pre-pregnancy weight status. Serum folate concentration decreased as pre-pregnancy BMI increased, as others have found [33]. Serum folate concentration was significantly higher in underweight women compared to overweight women in our study (23.4 ± 1.7 *vs.* 17.0 ± 1.8 ng/mL); the mean values in overweight women was within the normal reference range of serum folate, 0.8 to 20.7 ng/mL for pregnant women [34]. Serum iron concentration was significantly higher in normal weight women compared to overweight women (86.2 ± 5.0 *vs.* 68.9 ± 3.0 ug/dL), all within the normal reference range of serum iron, 30 to 193 ug/dL for pregnant women [34]. Because mean serum folate and iron concentrations were all within normal reference ranges in all pre-pregnancy weight status groups, the differences may not be clinically relevant.

This study showed that diet quality during pregnancy measured using HEI-2010 was inversely associated with increasing pre-pregnancy BMI. Our study findings are in agreement with previous research results from Greece [12] and the United States [11]. In a cross-sectional study of Greek women [12], those who were underweight or had a normal pre-pregnancy BMI had a better diet quality as assessed by HEI-2005 compared to those who had an obese pre-pregnancy BMI. Consistent with this finding, in a prospective cohort study in North Carolina, pre-pregnancy BMI was inversely associated with diet quality, as assessed by the DQI-P [11]. Women who were obese before pregnancy had 76% increased odds of falling into the lowest tertile of diet quality indicated by DQI-P (mean 42, standard deviation 7.2) than those who were underweight before pregnancy. The major difference in our study compared to these two studies [11,12] is that we used the most current index (HEI-2010) while others used older versions, HEI-2005 and DQI-P. The updated HEI-2010 was chosen to assess the diet quality of pregnant women in the present study, for it reflects the most current 2010 Dietary Guidelines for Americans with key changes, such as the additional recommendations for seafood (fish and shellfish) and plant proteins, polyunsaturated and monounsaturated fatty acids, and limitations on refined grains [35]. 

In our study, the four most lacking components based on HEI-2010 scores for pregnant women’s diets were greens and beans, whole fruit, whole grains, and seafood and plant proteins. Strategies are needed to recommend greater consumption of greens and beans, whole fruit, whole grains, and seafood and plant proteins among pregnant women to improve overall diet quality. Food pricing strategies such as subsidies on fruits and vegetable and imposing taxes on carbonated drinks were associated with better diet quality and healthier food choices [36].

In our study, pregnant women who were older, of another race including the multi-racial group, married, non-smokers, and/or who had high income and high education levels had better diet quality. Our results confirm previous findings that pregnant women with advanced maternal age [37,38,39], high income [37], and high education [38,39] consumed diets of better quality. There are inconsistent findings for the association between race/ethnicity and diet quality during pregnancy. In our study, we found that non-Hispanic black pregnant women demonstrated the lowest HEI-2010 score compare to other race groups. Rifas-Shiman *et al.* [38] reported that African-American pregnant women had similar Alternate HEI-Pregnancy scores assessed in the second trimester of pregnancy compared to other race/ethnicity groups (59.4 ± 10.7 *vs.* 61.0 ± 10.0, respectively) in the prospective cohort study, Project Viva, after controlling for education and age. Bodnar *et al.* [37] also found no significant ethnic/race differences in mean DQI-P score measured in the second trimester of pregnancy among pregnant women who participated in the Pregnancy, Infection, and Nutrition study. This contradictory finding may be due to a different categorization of race/ethnicity groups. The Pregnancy, Infection, and Nutrition study [37] categorized race/ethnicity into white and black only, and Project Viva study [38] categorized race/ethnicity into black/African American, other, and white as the majority of the study population (72%). Our study stratified race/ethnicity into Mexican American or other Hispanic, non-Hispanic white, non-Hispanic Black, and other including multi-racial groups with even distributions across the race/ethnicity categories.

Dietary patterns during pregnancy have been associated with pregnancy complications and birth outcomes. In a prospective cohort study in Sweden [40], pregnant women who adhered to a “prudent” or “traditional” dietary pattern during pregnancy, characterized by high intake of vegetables, fruit, whole grains, and fish, were at lower risk of preterm birth. In a cross-sectional study of U.S. pregnant women [41], high consumption of added sugars and low consumption of fruits and vegetables during pregnancy were associated with increased risk for gestational diabetes mellitus. Maternal nutrition plays an important role in pregnancy complications and birth outcomes.

There are several limitations of this study. Due to cross-sectional study design in the NHANES, a cause–effect relationship cannot be made. The study focused on generating snapshots of the diet quality derived from foods and nutrients, and this information may not be adequate to represent the usual dietary intake of pregnant women. In assessing diet intake, one 24-h recall used in this study may not accurately estimate the habitual dietary intake of an individual. In addition, dietary underreporting by overweight and obese women could have resulted in obscured or confounded results in terms of the relationship between diet and weight status [42]. Dietary supplement use information was assessed using the Dietary Supplement Questionnaire as a part of the 24-h dietary recall interviews. The Dietary Supplement Questionnaire was used to collect information on the participant’s use of vitamins and minerals over the past 30 days. A limited number of pregnant women self-reported their use of dietary supplement. The association between biomarker concentrations and dietary supplement use could have been influenced by the weight status of the responders. Despite these limitations, the study has several strengths. First, we used a validated and reliable index, HEI-2010 [21], to assess the diet quality of representative U.S. pregnant women in addition to various maternal diet-related biomarkers and intake of supplement across the categories of pre-pregnancy weight status. Second, the study was based on representative U.S. pregnant women incorporating diverse groups of pregnant women in different months of pregnancy. Third, although the study used self-reported pre-pregnancy weight status, we previously validated self-reported pre-pregnancy weight status based on self-reported height and weight before pregnancy, and it was found to be valid [18]. Lastly, we were able to control for important maternal sociodemographic characteristics, such as smoking status and physical activity level during pregnancy, that may influence the relationship between pre-pregnancy weight status and diet quality.

## 5. Conclusions

In conclusion, pre-pregnancy weight status was inversely associated with diet quality and maternal nutritional biomarkers such as serum folate and iron concentrations during pregnancy. The association of pre-pregnancy weight status and diet quality remained significant even after controlling for maternal sociodemographic characteristics and physical activity during pregnancy. Given the increasing prevalence of overweight and obesity of reproductive aged women in the U.S. [43] and the negative effect of poor maternal nutrition on adverse birth outcomes, nutrition education and interventions with an emphasis to increase intake of whole grains, whole fruits, and seafood and plant proteins need to be targeted towards those women entering pregnancy as overweight and obese. Strategies to increase overall diet quality during pregnancy need to be further explored, such as subsidies on healthy foods such as fruits and vegetables along with nutrition assistance programs tailored for pregnant women. Future studies are warranted to explore the combined effect of pre-pregnancy weight status and maternal nutritional status during pregnancy on pregnancy complications and birth outcomes.

## Figures and Tables

**Table 1:** The Healthy Eating Index (HEI)-2010 components and standards.

Component	Maximum Points	Standard for Maximum Score	Standard for Minimum Score of Zero
HEI-2010 ^a^			
Adequacy:			
Total Fruit ^b^	5	≥0.8 cup equivalent/1000 kcal	No Fruit
Whole Fruit ^c^	5	≥0.4 cup equivalent/1000 kcal	No Whole Fruit
Total Vegetables ^d^	5	≥1.1 cup equivalent/1000 kcal	No Vegetables
Greens and Beans ^d^	5	≥0.2 cup equivalent/1000 kcal	No Dark Green Vegetables or Beans or Peas
Whole Grains	10	≥1.5 ounce equivalent/1000 kcal	No Whole Grains
Dairy ^e^	10	≥1.3 cup equivalent/1000 kcal	No Dairy
Total Protein Foods ^f^	5	≥2.5 ounce equivalent/1000 kcal	No Protein Foods
Seafood and Plant Proteins ^f,g^	5	≥0.8 ounce equivalent/1000 kcal	No Seafood or Plant Proteins
Fatty Acids ^h^	10	(PUFAs + MUFAs)/SFAs > 2.5	(PUFAs + MUFAs)/SFAs ≤ 1.2
Moderation:			
Refined Grains	10	≤1.8 ounce equivalents/1000 kcal	≥4.3 oz equivalent/1000 kcal
Sodium	10	≤1.1 g/1000 kcal	≥2.0 g per 1000 kcal
Empty Calories ^i^	20	≤19% of energy	≥50% of energy

Source: Adapted from: Guenther PM *et al.* [21]. ^a^ Intakes between the minimum and maximum standards are scored proportionately. ^b^ Includes fruit juice. ^c^ Includes all forms except juice. ^d^ Includes any beans and peas not counted as Total Protein Foods. ^e^ Includes all milk products, such as fluid milk, yogurt, and cheese, and fortified soy beverages. ^f^ Beans and peas are included here (and not with vegetables). ^g^ Includes seafood, nuts, seeds, soy products (other than beverages) as well as beans and peas counted as Total Protein Foods. ^h^ Ratio of polyunsaturated fatty acids (PUFAs) and monounsaturated fatty acids (MUFAs) to saturated fatty acids (SFAs). ^i^ Calories from solid fats, alcohol, and added sugars; threshold for counting alcohol is >13 g/1000 kcal.

**Table 2:** The mean Healthy Eating Index (HEI)-2010 scores by maternal characteristics (*n* = 795).

	*n*	Wt’d ^1^ %	Mean HEI-2010	SEM ^2^
Age				
≤25	355	37.9	45.7 *	0.9
26–35 (reference)	377	48.4	52.0	1.4
≥35	63	13.7	59.5 *	2.3
Race/ethnicity				
Mexican American or other Hispanic	272	22.6	53.5 *	1.2
Non-Hispanic white (reference)	317	51.7	50.6	1.4
Non-Hispanic black	152	17.7	53.1 *	1.2
Other including multi-racial	54	8.0	59.8 *	2.7
Family Poverty Income Ratio				
≤1.85	427	43.6	47.7 *	1.1
1.85–4	185	26.1	50.3	1.5
>4 (reference)	183	30.3	55.1	1.9
Education Level				
≤11th grade	288	23.4	46.2 *	1.3
High school grade	143	17.8	48.5	1.4
Above college (reference)	364	58.7	53.1	1.4
Marital Status (*n* = 794)				
Married (reference)	466	63.9	53.5	1.3
Widowed/divorced/separated/living with a partner	152	15.9	47.7	1.6
Single	176	20.2	44.1 *	1.5
Parity (*n* = 488)				
None (reference)	37	7.0	43.8	2.7
1–5	446	91.3	50.2	1.3
≥6	5	1.7	50.1	1.2
Trimester of Pregnancy (*n* = 640)				
1st trimester (reference)	136	26.6	48.4	2.6
2nd trimester	257	34.6	52.6	2.0
3rd trimester	247	38.8	51.6	1.6
Physical activity (*n* = 526)				
No activity	107	30.8	51.6	1.5
0 to <500 MET ^3^-min/week	217	34.6	52.0	2.1
500 to <1000 MET-min/week	91	17.4	54.2	2.8
≥1000 MET-min/week (reference)	111	17.2	50.9	2.5
Smoking ^4^				
Non-smoker (reference)	705	91.0	51.8	1.0
Smoker	90	9.0	38.7 *	1.6
Total	795	100.0	50.7	0.9

^1^ Wt’d %: Weighted %. Sample weights are created in NHANES to account for the complex survey design (including oversampling of some subgroups), survey non-responses, and post-stratification. When a sample is weighted in NHANES, it is representative of the U.S. civilian non-institutionalized census population. ^2^ SEM: Standard error of the mean. ^3^ Total MET-min/week from self-reported leisure-time physical activities. * Significant at *p* < 0.001, using analysis of variance with Bonferroni correction. ^4^ Smoking status was defined by a serum cotinine concentration (non-smoker: ≤10 mg/L; smoker >10 mg/L). * Significant difference from the reference group (*p* < 0.05).

**Table 3:** The Healthy Eating Index (HEI)-2010 scores ^1^ across categories of pre-pregnancy weight status (*n* = 795).

		Pre-Pregnancy Weight Status				
	Maximum Points	Underweight (*n* = 124)	Normal (*n* = 343)	Overweight (*n* = 173)	Obese (*n* = 155)	*p* for Trend
Overall HEI-2010 ^2^	100	54.7 ± 2.1 ^a,b^	55.2 ± 1.6 ^b^	52.3 ± 2.8 ^a,b^	48.8 ± 2.0 ^a^	0.0074
Total Vegetables	5	3.4 ± 0.3	3.2 ± 0.2	3.2 ± 0.3	2.9 ± 0.4	0.28
Greens and Beans	5	1.3 ± 0.3	2.0 ± 0.3	1.3 ± 0.3	1.6 ± 0.4	0.71
Total Fruit	5	3.2 ± 0.3	3.1 ± 0.3	2.5 ± 0.4	2.4 ± 0.3	0.02
Whole Fruit	5	2.9 ± 0.4	2.8 ± 0.3	2.4 ± 0.4	2.0 ± 0.3	0.01
Whole Grains	10	2.2 ± 0.5	2.4 ± 0.4	2.3 ± 0.7	1.6 ± 0.5	0.66
Dairy	10	6.2 ± 0.5	6.0 ± 0.4	5.6 ± 0.6	6.5 ± 0.5	0.98
Total Protein Foods	5	4.3 ± 0.3	4.0 ± 0.2	4.3 ± 0.2	4.2 ± 0.3	0.64
Seafood and Plant Proteins	5	1.6 ± 0.3	2.2 ± 0.2	1.7 ± 0.4	1.8 ± 0.4	0.99
Fatty Acids	10	4.2 ± 0.7	4.2 ± 0.5	4.9 ± 0.9	3.9 ± 0.6	0.91
Sodium	10	4.3 ± 0.6 ^a,b^	5.4 ± 0.4 ^b^	4.0 ± 0.5 ^a,b^	3.7 ± 0.6 ^a^	0.04
Refined Grains	10	6.1 ± 0.6	6.3 ± 0.4	5.6 ± 0.5	5.7 ± 0.5	0.53
Empty Calories ^3^	20	14.9 ± 1.2	13.7 ± 0.8	14.5 ± 1.0	12.4 ± 0.9	0.07

^1^ Adjusted for maternal age, race/ethnicity, family poverty income ratio, education, marital status, smoking status and physical activity. ^2^ The HEI-2010 total score (range: 0 to 100) was computed by adding 12 component that sum to a maximum score of 100 [31]. The HEI-2010 includes 12 components, nine of which assess adequacy of diet including total vegetables, greens and beans, total fruit, whole fruit, whole grains, dairy, total protein, foods, seafood and plant proteins, as well as the fatty acid ratio. The remaining three, sodium, refined grains, empty grains, and empty calories, assess dietary components that should be consumed in moderation. The HEI-2010 scores use standards that are based on 1000 kcal. Values are weighted mean ± SEM (standard error of the mean). Different letters denote statistically a significant difference across pre-pregnancy weight status groups, *p* < 0.05 (Bonferroni-adjusted *p* < 0.0125). ^3^ Empty calories refer to calories from solid fats, alcohol, and added sugars; threshold for counting alcohol is 0.28 g/day. *p* for trend was calculated by treating pre-pregnancy weight status (BMI, kg/m^2^) as a continuous variable.

**Table 4:** Dietary intake and diet-related biomarkers during pregnancy across categories of pre-pregnancy weight status.

	Pre-pregnancy Weight Status							
	Underweight		Normal		Overweight		Obese	
	*n*	Mean (SEM)	*n*	Mean (SEM)	*n*	Mean (SEM)	*n*	Mean (SEM)
Dietary Intake								
Energy Intake (kcal/day)	124	2139.5 (99.8)	343	2245.6 (82.9)	173	2153.8 (55.5)	155	2326.6 (88.0)
%Energy Carbohydrate	124	54.6 (1.4)	343	53.3 (1.0)	173	52.6 (1.3)	155	50.6 (1.4)
%Energy Protein	124	15.4 (0.6)	343	14.5 (0.4)	173	15.8 (0.5)	155	15.3 (0.4)
%Energy Fat	124	31.5 (1.4)	343	33.5 (0.8)	173	32.8 (1.1)	155	34.8 (1.1)
Folate, DFE (mcg/day)	124	659.0 (69.3)	343	627.6 (40.3)	173	675.7 (70.8)	155	558.2 (36.6)
Folate, DFE (mcg)/1000 kcal	124	319.5 (27.4) ^b^	343	282.2 (15.0) ^a,b^	173	319.4 (36.1) ^a,b^	155	246.1 (15.9) ^a^
Iron (mg/day)	124	18.1 (1.2)	343	17.2 (0.9)	173	19.4 (1.6)	155	15.8 (0.8)
Iron (mg)/1000 kcal	124	8.9 (0.5) ^b^	343	7.8 (0.3) ^a,b^	173	9.1 (0.8) ^a,b^	155	6.9 (0.3) ^a^
Calcium (mg/day)	124	1139.8 (63.2)	343	1132.2 (62.2)	173	1060.1 (63.6)	155	1131.9 (89.1)
Calcium (mg)/1000 kcal	124	568.4 (41.4)	343	507.6 (22.4)	173	516.6 (42.0)	155	489.3 (30.6)
Biomarkers								
Serum folate (ng/mL)	115	23.4 (1.7) ^b^	321	19.1 (0.7) ^a,b^	158	17.0 (0.8) ^a^	143	17.4 (1.8) ^a,b^
Ferritin (ng/mL)	72	44.5 (9.2)	321	34.7 (3.8)	158	35.1 (3.7)	143	44.5 (6.4)
Iron (ug/dL)	72	79.4 (9.4) ^a,b^	322	86.2 (5.0) ^b^	158	68.9 (3.0) ^a^	143	72.2 (5.5) ^a,b^
Dietary Supplement Intake ^1^								
Folic acid (mcg/day)	29	838.8 (90.3)	40	781.8 (36.8)	13	1186.2 (181.7)	15	922.7 (105.0)
Folate, DFE (mcg/day)	29	1426.0 (153.5)	40	1329.0 (62.5)	13	2016.6 (308.9)	15	1568.5 (178.6)
Iron (mg/day)	28	29.3 (2.6)	37	41.1 (5.4)	12	81.7 (28.0)	17	30.2 (1.8)
Zinc (mg/day)	28	19.5 (2.1)	38	19.9 (1.2)	11	22.9 (4.8)	12	16.4 (2.8)
Calcium (mg/day)	28	346.7 (77.5)	38	294.5 (53.9)	10	253.0 (61.9)	17	544.0 (170.1)

Values are weighted mean ± SEM. Different letters denote statistically a significant difference across pre-pregnancy weight status groups, *p* < 0.05 (Bonferroni-adjusted *p* < 0.0125). ^1^ information on dietary supplement intake data was available for the NHANES 2007–2012 periods, but not for the NHANES 2003–2006.

**Table 5:** Associations between the lowest Healthy Eating Index (HEI)-2010 tertile and pre-pregnancy weight status categories.

	HEI-2010 Scores Tertile ^1^ 3 (Reference) *vs.* Tertile 1		
	Crude	Model 1	Model 2
Pre-pregnancy Weight Status			
Obese	5.0 (2.2–11.4) *	3.7 (1.7–8.2) *	5.4 (2.0–14.5) *
Overweight	2.6 (1.1–6.4) *	2.8 (1.2–6.6) *	3.8 (1.2–11.7) *
Normal weight	1.9 (0.9–4.2)	1.7 (0.8–3.4)	1.9 (0.8–4.6)
Underweight	1.0	1.0	1.0

Model 1: Adjusted for age, race/ethnicity, family poverty income ratio, education level, marital status, and smoking status (*n* = 794). Model 2: Adjusted for model 1 + physical activity level (*n* = 525). ^1^ Tertiles 1, 2, and 3 represent pregnant women in the lowest, intermediate and highest thirds of the HEI-2010 score, respectively. Mean (±SEM) of tertiles 1, 2, and 3 are 33.4 (±0.5), 48.5 (±0.3), and 66.5 (±0.9), respectively. ^2^ Total MET-min/week from self-reported leisure-time physical activities. * *p* < 0.05.
